# Supplementary material for: Dissecting super-enhancer hierarchy based on chromatin interactions
Source: Nat Commun. 2018 Mar 5;9:943. doi: 10.1038/s41467-018-03279-9 (PMC5838163; doi:10.1038/s41467-018-03279-9)
Supplement: Supplementary file 1 — Supplementary Information [file 41467_2018_3279_MOESM1_ESM.pdf]

Supplementary Information

Huang *et al.* Dissecting super-enhancer hierarchy based on chromatin interactions.

Supplementary Figure 1. Definition of hierarchical SEs and hub enhancers using chromatin interactions in K562 and GM12878 cells.

Supplementary Figure 2. Hierarchical and non-hierarchical SEs in GM12878 cells.

Supplementary Figure 3. Chromatin landscapes around hub enhancers in GM12878 cells.

Supplementary Figure 4. CTCF binding at hub enhancers within hierarchical SEs in GM12878 cells.

Supplementary Figure 5. Identification of enhancer-promoter mapping in K562 cells.

Supplementary Figure 6. Enrichment of genetic variants associated with cell-type specific gene expression and diseases in hub enhancers in GM12878 cells.

Supplementary Figure 7. Enrichment of genetic variants associated with cell-type specific expression and diseases in K562 and GM12878 cells.

Supplementary Figure 8. Hierarchical SEs and hub enhancers identified in IMR90, HMEC and HUVEC cells.

Supplementary Figure 9. Comparative analysis of Hi-C and ChIA-PET based hub enhancers.

Supplementary Figure 10. *In situ* analysis of the functional requirement of hub vs non-hub enhancers.

Supplementary Table 1. List of primer and sgRNA sequences used in this study.

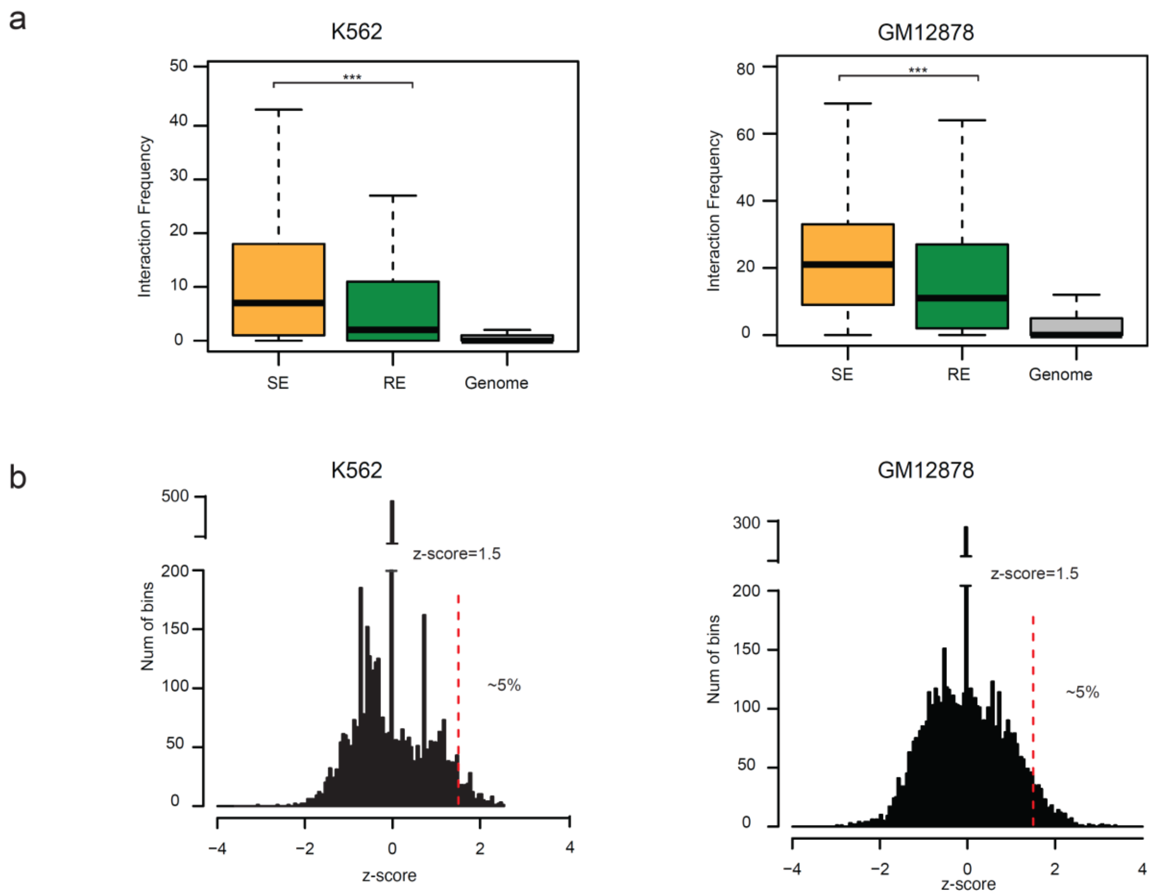

c

K562

H-score	Hierarchical SEs	Hub enhancers
1.25	313	750
1.50	198	444
1.75	114	246

GM12878

H-score	Hierarchical SEs	Hub enhancers
1.25	457	1052
1.50	286	606
1.75	153	316

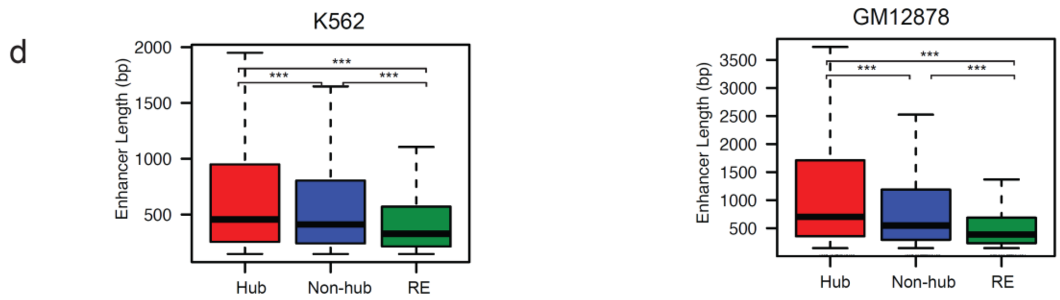

Supplementary Figure 1. Definition of hierarchical SEs and hub enhancers using chromatin interactions in K562 and GM12878 cells.

(**a**) Chromatin interactions frequency for 5kb bins overlapping with SEs (yellow), REs (green), using randomly selected genome 5kb bins as control (gray) in K562 and GM12878 cells. In box

plots, the center line represents the median, the box limits represent the 25th and 75th percentiles and the whiskers represent the 5 and 95th percentiles. *P* values were calculated using Student's t-test. **P* < 0.05; ***P* < 0.01; ****P* < 0.001, n.s. not significant.

(**b**) Distribution of z-score of 5kb bins in all SEs. The dashed line represents the threshold value of H-score = 1.5, which roughly corresponds to the 95th percentile of z-scores.

(**c**) The number of hierarchical SEs and hub enhancers identified using various thresholds of H-score.

(**d**) Distribution of the enhancer length in three enhancer groups, hub, non-hub and regular enhancer. In box plots, the center line represents the median, the box limits represent the 25th and 75th percentiles and the whiskers represent the 5 and 95th percentiles. *P* values were calculated using Student's t-test. **P* < 0.05; ***P* < 0.01; ****P* < 0.001, n.s. not significant.

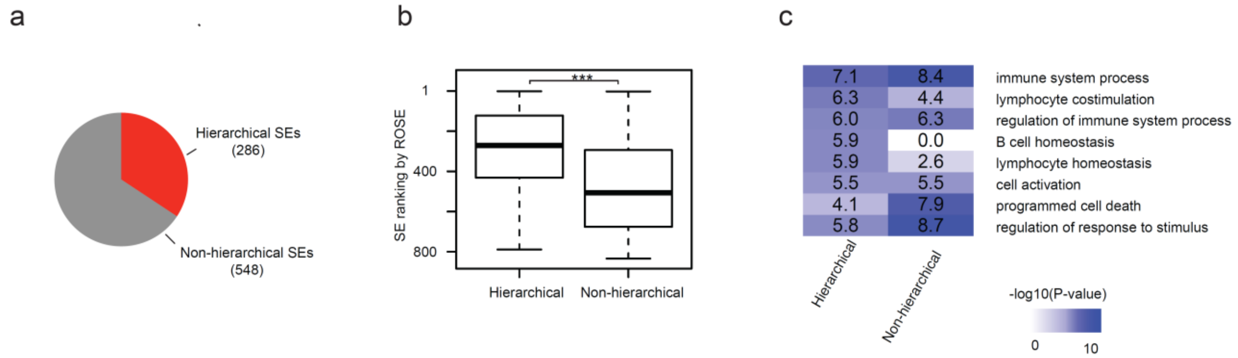

Supplementary Figure 2. Hierarchical and non-hierarchical SEs in GM12878 cells.

(**a**) Proportion of hierarchical and non-hierarchical SEs.

(**b**) The ROSE ranking of hierarchical and non-hierarchical SEs. In box plots, the center line represents the median, the box limits represent the 25th and 75th percentiles and the whiskers represent the 5 and 95th percentiles. *P* value was calculated using Wilcoxon rank-sum test. *P < 0.05; **P < 0.01; ***P < 0.001, n.s. not significant.

(**c**) GREAT functional analysis of hierarchical and non-hierarchical SEs. The significance level of each enriched category was indicated in the heatmap.

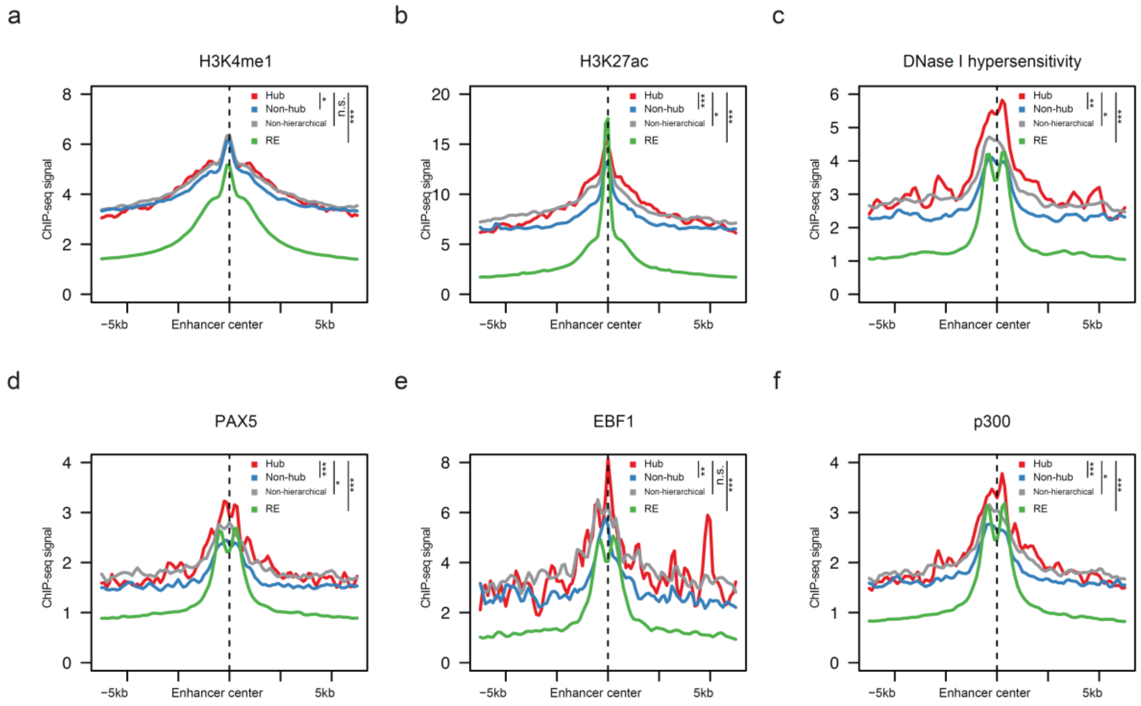

Supplementary Figure 3. Chromatin landscapes around hub enhancers in GM12878 cells.

(**a-f**) Spatial distribution of chromatin marks centered by enhancers in four groups, hub (n=606), non-hub enhancers (n=3704), enhancers in non-hierarchical SEs (n=4653) and regular enhancers (27312): H3K4me1 (**a**), H3K27ac (**b**), DNase I hypersensitivity (**c**), master regulators PAX5 (**d**) and EBF1 (**e**), and coactivator p300 (**f**).

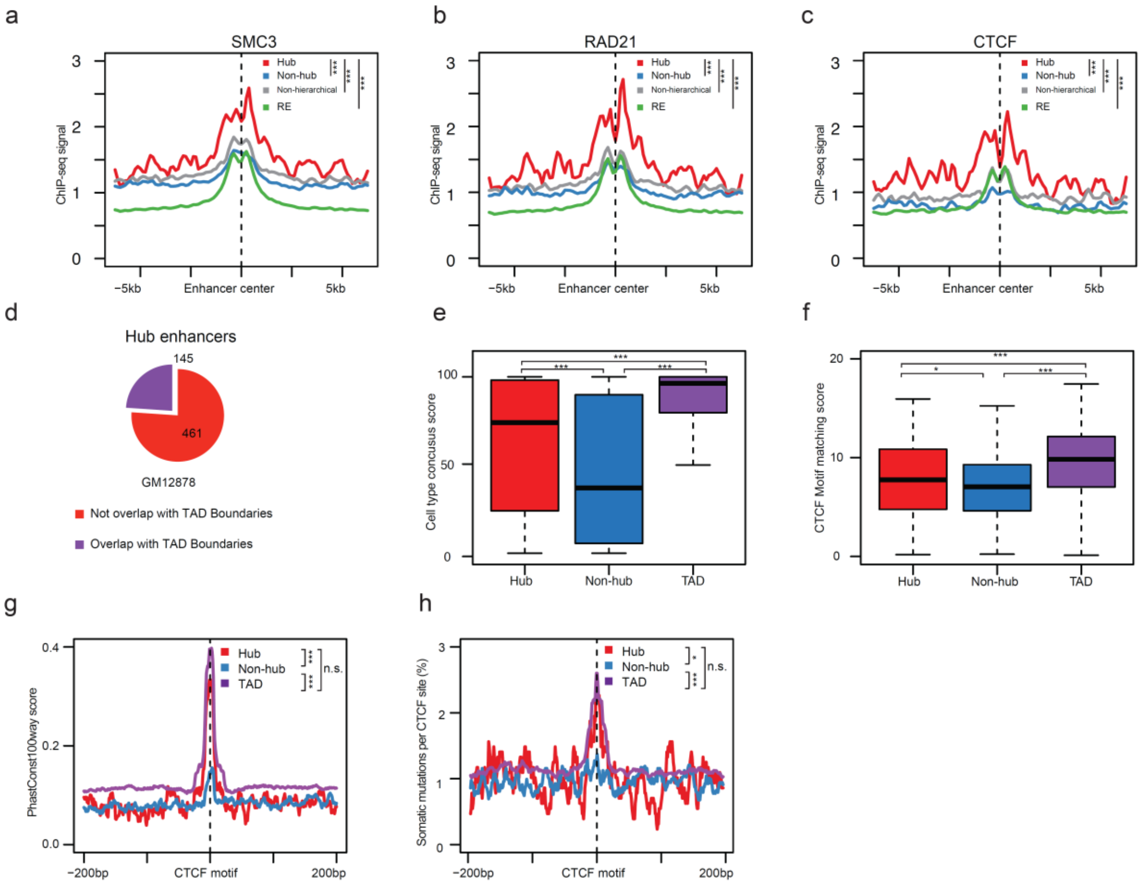

Supplementary Figure 4. CTCF binding at hub enhancers within hierarchical SEs in GM12878 cells.

(**a**-**c**) Spatial distribution of two cohesin components SMC3, RAD21 (**a**,**b**) and CTCF (**c**), centered by enhancers in four groups.

(**d**) Percentage of hub enhancers with (purple) or without (red) overlapping with TAD boundaries collected from[15]. The CTCF ChIP-seq peaks/motif-sites associated with hub enhancers overlapping with TAD boundaries were excluded for analysis in (**e-h**).

(**e-f**) Distribution of the CTCF binding consensus across cell types (**e**) and CTCF-motif-matching score (**f**) of CTCF peaks: hub (red), non-hub enhancers (blue) and TAD boundaries (purple). For each CTCF peak in GM12878, the consensus score (y-axis) was quantified as the percentage of cell types containing the same CTCF peak. In box plots, the center line represents the median, the box limits represent the 25th and 75th percentiles and the whiskers represent the 5 and 95th percentiles. *P* values were calculated using Student's t test. **P* < 0.05; ***P* < 0.01; ****P* < 0.001, n.s. not significant.

(**g**) Distribution of the sequence conservation scores around CTCF motif sites. The sitepro plots were centered by CTCF motif sites. *P* values were calculated using Student's t-test based on the PhastConst100way score (y-axis) within CTCF motif sites. **P* < 0.05; ***P* < 0.01; ****P* < 0.001, n.s. not significant.

(**h**) Distribution of the somatic mutation rate in cancers around CTCF motif sites. The sitepro plots were centered by CTCF motif sites with 10bp smoothing window. *P* values were calculated using Fisher's exact test based on overlap between CTCF motif sites and somatic mutation sites. **P* < 0.05; ***P* < 0.01; ****P* < 0.001, n.s. not significant.

a b c

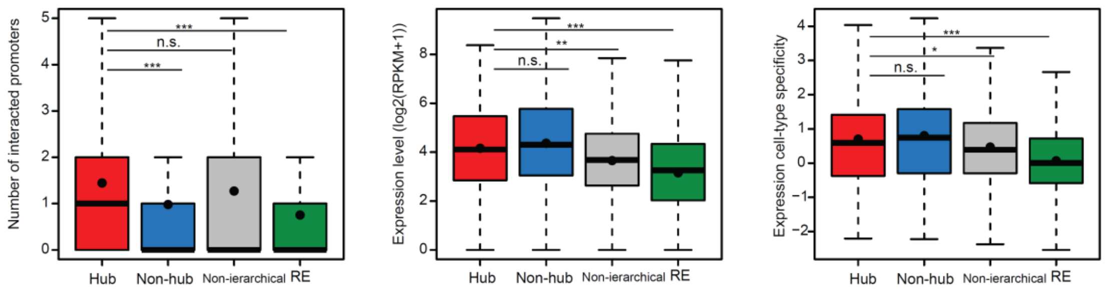

Supplementary Figure 5. Identification of enhancer-promoter mapping in K562 cells.

(**a**) The number of enhancer-promoter mappings for enhancers in four groups. The dot within the boxplot represent its' mean. *P* values were calculated using Student's t-test. *$P < 0.05$; **$P < 0.01$; ***$P < 0.001$, n.s. not significant.

(**b**) The expression level of mapped target genes for enhancers. The dot within the boxplot represent its' mean. *P* values were calculated using Student's t-test. *$P < 0.05$; **$P < 0.01$; ***$P < 0.001$, n.s. not significant.

(**c**) The expression cell-type specificity of mapped target genes for enhancers. In box plots, the dot represents the mean, the center line represents the median, the box limits represent the 25th and 75th percentiles and the whiskers represent the 5 and 95th percentiles. *P* values were calculated using Student's t-test. *$P < 0.05$; **$P < 0.01$; ***$P < 0.001$, n.s. not significant.

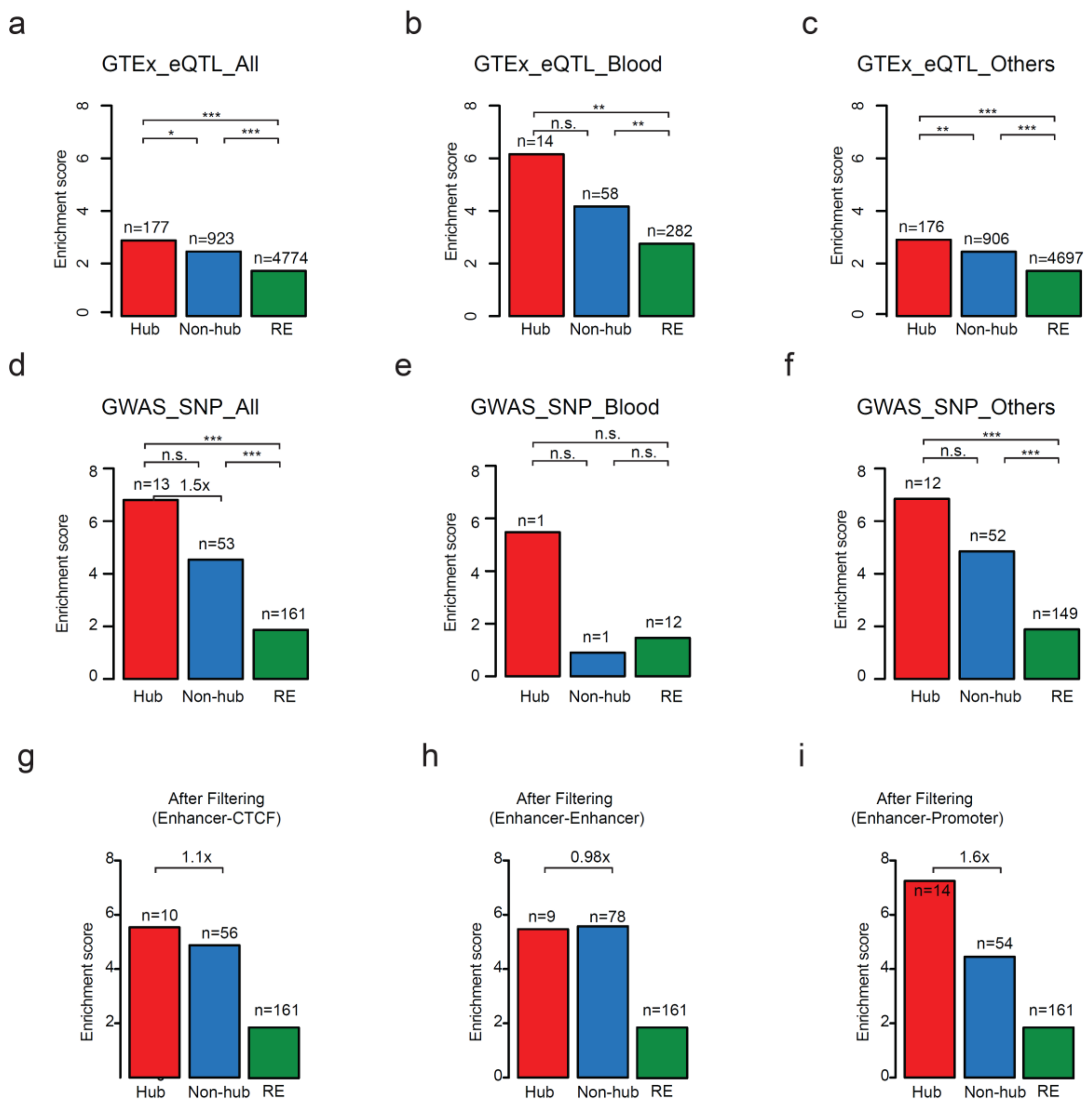

Supplementary Figure 6. Enrichment of genetic variants associated with cell-type specific gene expression and diseases in hub enhancers in GM12878 cells.

(**a-c**) Enrichment of the eQTLs curated in GTEx in the enhancers in three groups, using randomly selected genome regions as control. The GTEx eQTL identified in all tissues (**a**) were separated into two subsets, identified in blood (**b**) or other tissues (**c**). The number of enhancers overlapped with eQTLs in each group was labelled on each bar. *P* values were calculated using Fisher's exact test. *$P < 0.05$; **$P < 0.01$; ***$P < 0.001$, n.s. not significant.

(**d-f**) Enrichment of the disease or traits-associated SNPs curated in GWAS catalog in the enhancers in three groups, using randomly selected genome regions as control. The GWAS SNPs associated all diseases/traits (**d**), were separated into two subsets, associated with blood-

related diseases/traits (**e**) or other traits (**f**). The number of enhancers overlapped with SNPs in each group was labelled on each bar. *P* values were calculated using Fisher's exact test. *$P < 0.05$; **$P < 0.01$; ***$P < 0.001$, n.s. not significant.

(**g-i**) Enrichment of GWAS SNPs in hub and non-hub enhancers, which were defined based on chromatin interactions after filtering a specific subtype of chromatin interactions, enhancer-CTCF (**g**), enhancer-enhancer (**h**), enhancer-promoter (**i**). The fold-change between hub and non-hub enhancers were labelled.

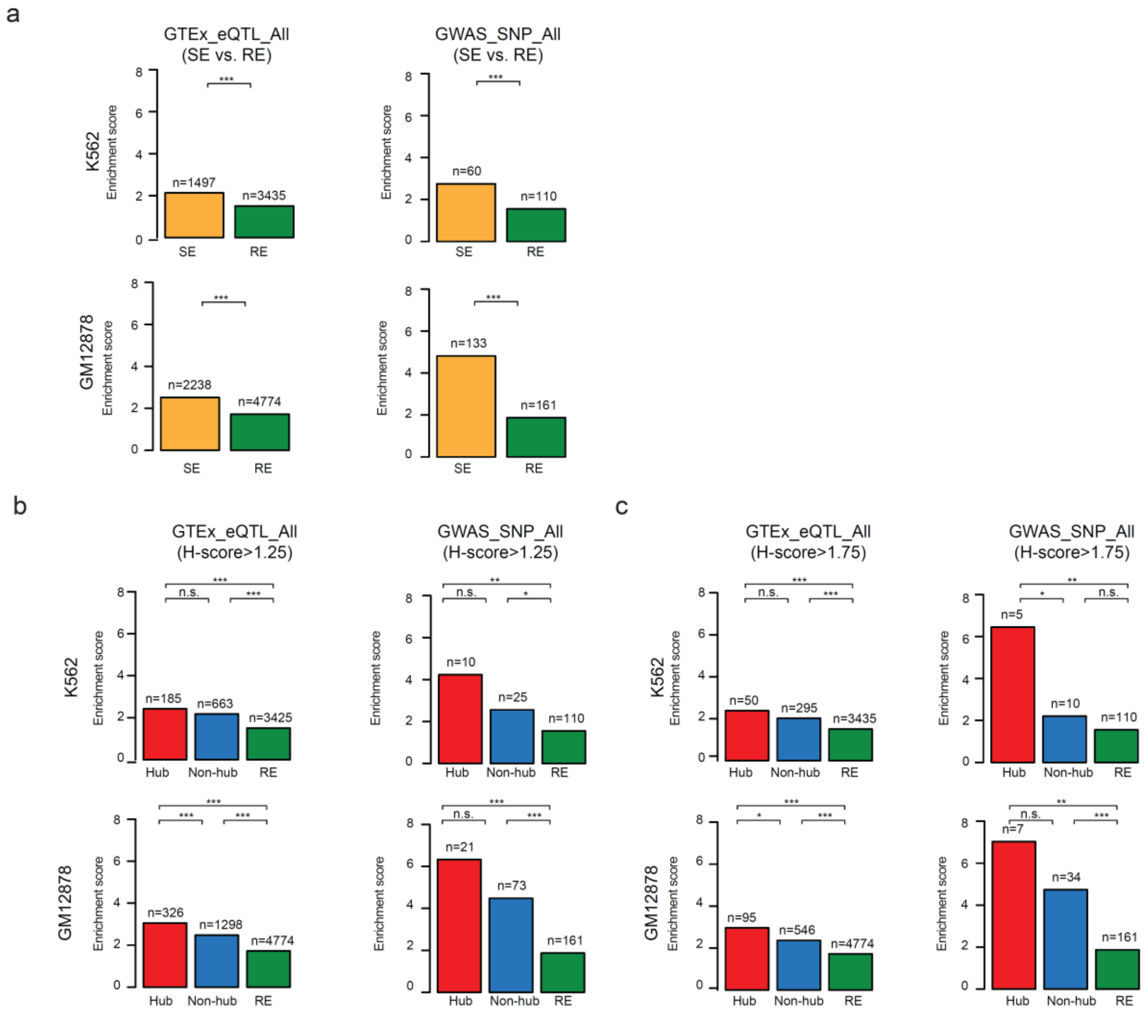

Supplementary Figure 7. Enrichment of genetic variants associated with cell-type specific expression and diseases in K562 and GM12878 cells.

(**a**) Enrichment of GTEx eQTL (left) and GWAS SNPs (right) in SEs and REs in K562 (upper) and GM12878(lower) cells. The number of enhancers overlapped in each group with eQTLs was labelled on each bar. *P* values were calculated using Fisher's exact test. **P* < 0.05; ***P* < 0.01; ****P* < 0.001, n.s. not significant.

(**b,c**) Enrichment of GTEx eQTL (left) and GWAS SNPs (right) in hub enhancers defined using the threshold of H-score >1.25 (**b**) or H-score >1.75 (**c**) in K562 (upper) and GM12878 (lower) cells. The number of enhancers overlapped in each group with eQTLs was labelled on each bar. *P* values were calculated using Fisher's exact test. **P* < 0.05; ***P* < 0.01; ****P* < 0.001, n.s. not significant.

a

Cell	SEs	Enhancers in SEs	Hierarchical SEs	Hub Enhancers	Hierarchical SEs (%)	Hub enhancers (%)
IMR90 (Human Fetal Lung Fibroblasts Cell)	805	18310	500	1829	62%	10%
HMEC (Human Mammary Epithelial Primary Cell)	1243	6757	334	397	27%	6%
HUVEC (Human Umbilical Vein Endothelial Primary Cell)	1114	5985	293	349	26%	6%

b

c

d

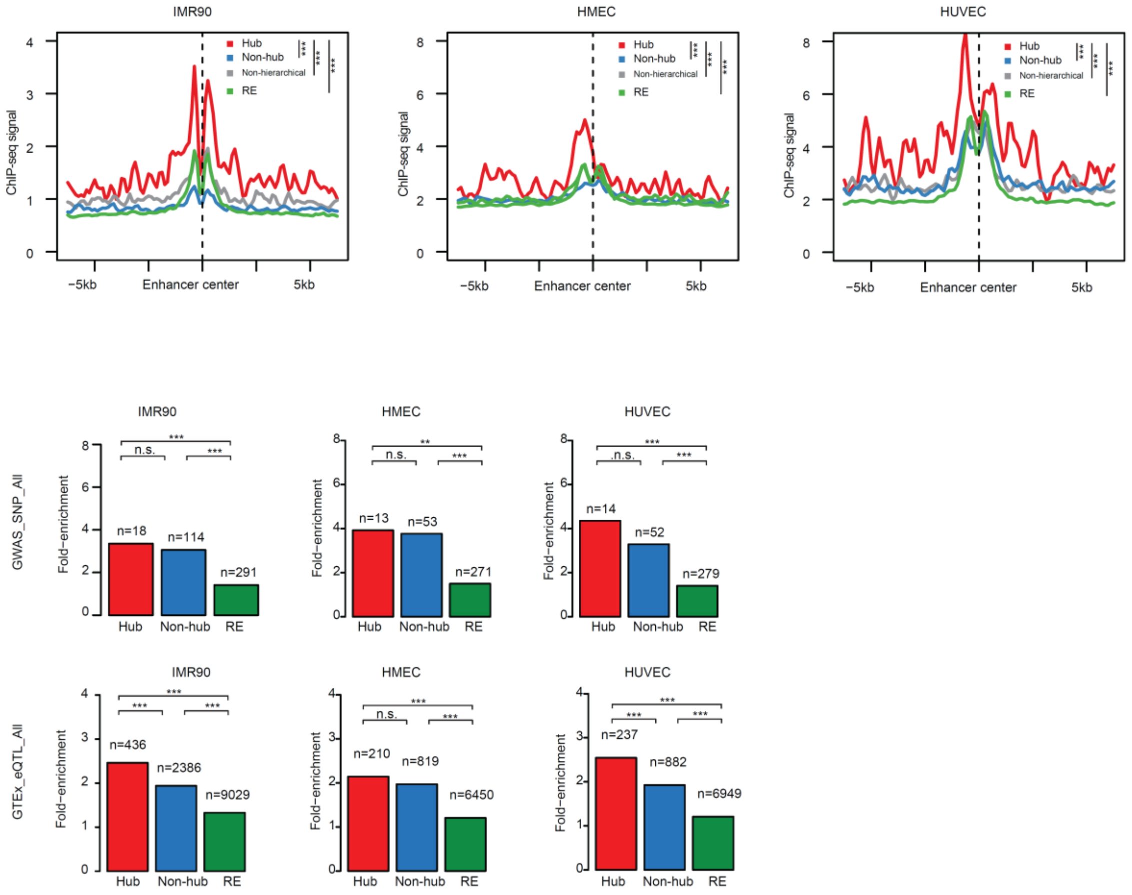

Supplementary Figure 8. Hierarchical SEs and hub enhancers identified in IMR90, HMEC and HUVEC cells.

(**a**) The number of hierarchical SEs and hub enhancers identified in IMR90, HMEC and HUVEC cells.

(**b**) The spatial distribution of ChIP signal of CTCF binding at four groups of enhancers.

(**c-d**) Enrichment of GWAS SNPs and GTEx eQTLs in the enhancers in three groups using randomly selected genome regions as control. The number of enhancers overlapped with SNPs or eQTLs in each group was labelled on each bar. *P* values were calculated using Fisher's exact test. *$P < 0.05$; **$P < 0.01$; ***$P < 0.001$, n.s. not significant.

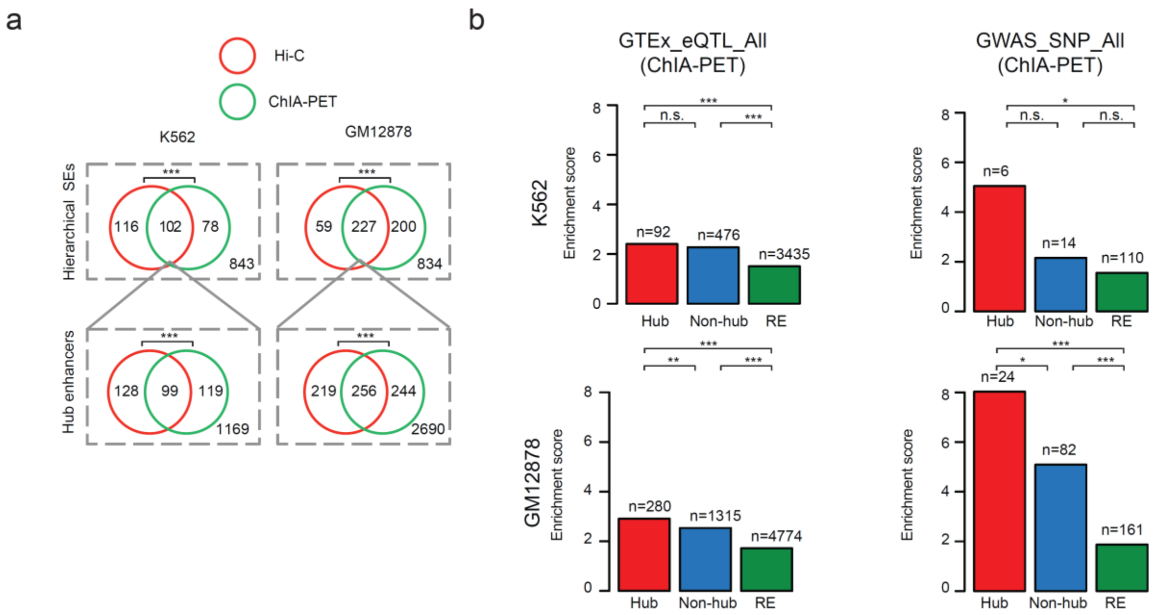

Supplementary Figure 9. Comparative analysis of Hi-C and ChIA-PET based hub enhancers.

(**a**) Overlap between the hierarchical SEs (left) or hub enhancers (right) identified using Hi-C and ChIA-PET dataset in K562 (upper) and GM12878 (lower) cells. *P* values were calculated using Fisher's exact test. **P* < 0.05; ***P* < 0.01; ****P* < 0.001, n.s. not significant.

(**b**) Enrichment of GTEx eQTL (left) or GWAS SNPs (right) in hub enhancers identified based on chromatin interactions detected by ChIA-PET. *P* values were calculated using Fisher's exact test. **P* < 0.05; ***P* < 0.01; ****P* < 0.001, n.s. not significant.

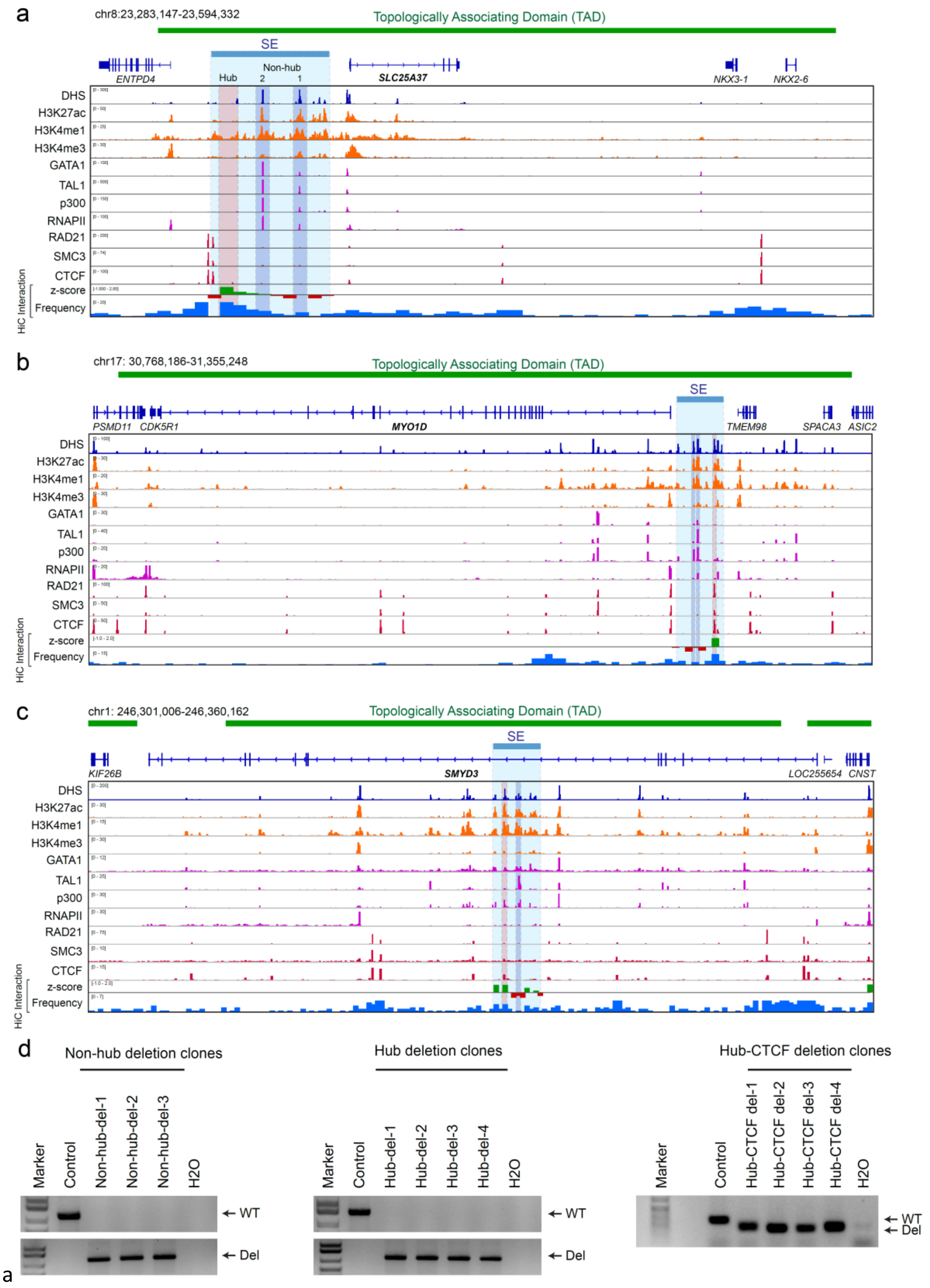

Supplementary Figure 10. *In situ* analysis of the functional requirement of hub vs non-hub enhancers.

(**a**) A genome browser view of the chromatin signatures and TF occupancy at the *SLC25A37* SE locus in K562 cells. The identified SE is depicted by the blue shaded area. The hub and non-hub enhancers are denoted by the red and blue shaded lines, respectively. The Hi-C chromatin interaction z-score and frequency at 5kb resolution is shown at the bottom.

(**b**) A zoomed-out view of the chromatin landscape and TF binding at the *MYO1D* SE locus in K562 cells.

(**c**) A zoomed-out view of the chromatin landscape and TF binding at the *SMYD3* SE locus in K562 cells.

(**d**) The representative genotyping results of CRISPR/Cas9-mediated knockout of hub, non-hub enhancers or the CTCF binding site within the hub enhancer at the *MYO1D* SE locus in K562 cells. Del: genotyping PCR primers for detection of enhancer deletion; WT: genotyping PCR primers for detection of the wild-type (or unmodified) region; Control: unmodified K562 cells.

Supplementary Table 1. List of primer and sgRNA sequences used in this study.

Name	*Forward*	*Reverse*	*Application*
MYO1D_Non-hub Enh1-sgRNA1	CACCGCTTATCTGTTCGTTCGTGTC	AAACGACACGAACGAACAGATAAGC	sgRNA oligos for CRISPRi
MYO1D_Non-hub Enh1-sgRNA2	CACCGTGAACTGATACACTAATTGC	AAACGCAATTAGTGTATCAGTTCAC	
MYO1D_Non-hub Enh2-sgRNA1	CACCGCATGTAGCAACATGTGATAC	AAACGTATCACATGTTGCTACATGC	
MYO1D_Non-hub Enh2-sgRNA2	CACCGCATTGGCACTCTCTGCCGTC	AAACGACGGCAGAGAGTGCCAATGC	
MYO1D_Hub Enh-sgRNA1	CACCGGCTAACGTTGAAGATTGCTG	AAACCAGCAATCTTCAACGTTAGCC	
MYO1D_Hub Enh-sgRNA2	CACCGGCACTTCAAAGAGTGGTCAC	AAACGTGACCACTCTTTGAAGTGCC	
SMYD3_Hub Enh-sgRNA1	CACCGGGACTGTTCCTCTCAAAAGT	AAACACTTTTGAGAGGAACAGTCCC	
SMYD3_Hub Enh-sgRNA2	CACCGGAAGTCCAGGTTATGACTGT	AAACACAGTCATAACCTGGACTTCC	
SMYD3_Non-hub Enh-sgRNA1	CACCGGTGAGCTTACCCGTGACTCC	AAACGGAGTCACGGGTAAGCTCACC	
SMYD3_Non-hub Enh-sgRNA2	CACCGCCTATCTATTCGTTGCAGTG	AAACCACTGCAACGAATAGATAGGC	
sgGal4	CACCGAACGACTAGTTAGGCGTGTA	AAACTACACGCCTAACTAGTCGTTC	
MYO1D_Non-hub Enh1-sgRNA1	CACCGGGGGCATGGTTTTGACAACC	AAACGGTTGTCAAAACCATGCCCCC	sgRNA oligos for KO
MYO1D_Non-hub Enh1-sgRNA2	CACCGGTTGTCGTTGTTGTTAACTC	AAACGAGTTAACAACAACGACAACC	
MYO1D_Non-hub Enh1-sgRNA3	CACCGACTGTTGCCTGGTAAATTAA	AAACTTAATTTACCAGGCAACAGTC	
MYO1D_Non-hub Enh1-sgRNA4	CACCGTGTCACCCAGAATGGAGTGC	AAACGCACTCCATTCTGGGTGACAC	
MYO1D_Hub Enh-sgRNA1	CACCGGGAGATGAGATACAGAGTAG	AAACCTACTCTGTATCTCATCTCCC	
MYO1D_Hub Enh-sgRNA2	CACCGGTAAAGCAGAATAGGGGCAT	AAACATGCCCCTATTCTGCTTTACC	
MYO1D_Hub Enh-sgRNA3	CACCGCCATTTTACAGTTGTCCCCC	AAACGGGGGACAACTGTAAAATGGC	
MYO1D_Hub Enh-sgRNA4	CACCGTCTCATTCCTTCGTCGCCAC	AAACGTGGCGACGAAGGAATGAGAC	
MYO1D_Hub CTCF-sgRNA1	CACCGTTTTAGGTTGTCAGCAGATC	AAACGATCTGCTGACAACCTAAAAC	
MYO1D_Hub CTCF-sgRNA2	CACCGCTGACAACCTAAAACCAAGA	AAACTCTTGGTTTTAGGTTGTCAGC	
MYO1D_Hub CTCF-sgRNA3	CACCGGCTAACGTTGAAGATTGCTG	AAACCAGCAATCTTCAACGTTAGCC	
MYO1D_Hub CTCF-sgRNA4	CACCGTCAACGTTAGCTCACAGATG	AAACCATCTGTGAGCTAACGTTGAC	
MYO1D_Non-hub Enh1-del	GCTGATATTGAACTCCTGACCTC	ATGGTGGTACATGCCTGTGGTC	genotyping primers for enhancer KO
MYO1D_Non-hub Enh1-WT	GCTGATATTGAACTCCTGACCTC	ATATACAGTGTCTGAGTGGCAAAGC	
MYO1D_Hub Enh-del	AAGTTGAAGAGAGAACGGGAGGTAG	CCCTGGCTCTGTTGTGAAATGTGG	
MYO1D_Hub Enh-WT	AAGTTGAAGAGAGAACGGGAGGTAG	CACGGAGTTGCTCTCTTGCTCTTC	
MYO1D_Hub CTCF-del	ACTCCGTGGTGGCCTATGTGATCC	TGCCTTCAGTCCACTTCTAGTTACC	
hMYO1D_RT	AAGGCAGACTTCGTGCTGATG	TAAGGGTTCACAGAAACGACG	RT-qPCR primers
hTMEM98_RT	TTCTGGCTTCGTTTGCAGC	CGTCCAGTTCTAACTCAGAGGG	
hSPACA3_RT	CCGGCATAGAAGCCAGGAG	TCACAACGACCGTAGAGCTTG	
hCDK5R1_RT	AGAACAGCAAGAACGCCAAG	CGGCCACGATTCTCTTCCA	
hPSMD11_RT	GCCTCCATCGACATCCTCC	GAGCTGCTTTAGCCTTGCTG	
hSMYD3_RT	CGCGTCGCCAAATACTGTAGT	CAAGAAGTCGAACGGAGTCTG	
hGAPDH_RT	ACCCAGAAGACTGTGGATGG	TTCAGCTCAGGGATGACCTT	
MYO1D_Non-hub Enh1	TGTCTGTTCTGATCTGGTTCTTCTC	CTACAAGGAATTGACCAGCCTGAC	ChIP-qPCR primers
MYO1D_Hub Enh	TTTAGAAGCAGTGGTGACACCCAG	GAGAATGGTGAGGGCTCTGATGC	
MYO1D Prom	TCTCGGGAAAGCGCAGCCTC	GGCAAGGCAGACTTCGTGCTGATG	
TMEM98 Prom	GCGGGTGCCGCAGCTTTGTTCTTG	GACCCAAGACCCTACCCGCTTC	
Ctrl	AAACCCACGTCCAGCACAGTGTC	AATAGCGGGTAAGGATGTAGACAGG	
